# Proposal of a new family *Pseudodiploösporeaceae* fam. nov. (*Hypocreales*) based on phylogeny of *Diploöspora longispora* and *Paecilomyces penicillatus*

**DOI:** 10.1080/21501203.2022.2143919

**Published:** 2022-11-24

**Authors:** Jingzu Sun, Shuang Yu, Yongzhong Lu, Hongwei Liu, Xingzhong Liu

**Affiliations:** a State Key Laboratory of Mycology, Institute of Microbiology, Chinese Academy of Sciences, No. 3 Park 1, Beichen West Road, Chaoyang District, 100101, Beijing, China; b School of Medical Devices, Shenyang Pharmaceutical University, 110016, Shenyang, China; c School of Traditional Chinese Materia Medica, Key Laboratory of Structure-Based Drug Design & Discovery, Ministry of Education, Shenyang Pharmaceutical University, 110016, Shenyang, China; d School of Food and Pharmaceutical Engineering, Guizhou Institute of Technology, 550003, Guiyang, China; e Department of Microbiology, College of Life Science, Nankai University, 300350, Tianjin, China

**Keywords:** Five new combinations, two new genera, one new family, fungal pathogen, mushroom disease

## Abstract

During a field survey of cultivated *Morchella* mushroom diseases, *Diploöspora longispora* and *Paecilomyces penicillatus*, causal agents of pileus rot or white mould disease were detected, which resulted in up to 80% of yield losses. Multi-locus phylogenic analysis revealed that the fungi were affiliated in a distinct clade in *Hypocreales*. We further constructed a phylogenetic tree with broader sampling in *Hypocreales* and estimated the divergence times. The *D. longispora* and *P. penicillatus* clades were estimated to have diverged from *Hypocreaceae* around 129 MYA and *Pseudodiploösporeaceae* fam. nov is herein proposed to accommodate species in this clade. Two new genera, i.e. *Pseudodiploöspora* and *Zelopaecilomyceswere,* were introduced based on morphological characteristics and phylogenic relationships of *Diploöspora* longispora and *Paecilomyces penicillatus*, respectively. Five new combinations – *Pseudodiploöspora cubensis, P. longispora, P. fungicola, P. zinniae*, and *Zelopaecilomyces penicillatus* – were proposed.

## Introduction

True morels (*Morchella, Morchellaceae, Pezizales, Ascomycota*) are one of the most popular edible mushrooms with a long history of consumption in Asia, Europe, and North America (Pilz et al. 2017; Liu et al. 2018). Because of their good taste, culinary qualities, and pharmacological performances in antitumor, anti-inflammatory, and antioxidant activities (Tietel and Masaphy 2018; Zhang et al. 2019), demands for morels have significantly increased in the market. In recent years, large-scale field cultivation of *Morchella* was successfully achieved in China (Liu et al. 2018). The morel cultivation area reached approximately 12,000 ha in the production season of 2021–2020 in China, with an economic value of over RMB 10 billion. However, with the rapid expansion of cultivation, diseases become a bottleneck for morel production, especially for diseases caused by fungi. Several common fungal diseases have been identified in the fruiting bodies of cultivated *Morchella*: stipe rot disease caused by the *Fusarium incarnatum–F. equiseti* species complex (Guo et al. 2016) and by *Purpureocillium lilacinum* (Masaphy, 2022), cobweb disease caused by *Hypomyces*/*Cladobotryum* species (Lan et al. 2020), white mould disease caused by *Paecilomyces penicillatus* (He et al. 2017) and pileus rot disease caused by *Diploöspora longispora* (He et al. 2018; Liu et al. 2018). Previous investigations showed that white mould diseases and pileus rot resulted in up to 80% of morel yield losses each year, which was attributed to a large number of conidia quickly spreading around the cultivation areas (Wang et al. 2020). However, the fungal pathogens were mainly identified based on sequence similarity of internal transcribed spacer gene region (ITS) but lacked convincing morphological evidence (Hyde et al. 2017, 2018; Liu et al. 2018). When did a blast of the ITS sequence of *D*. *longispora*, Tanney et al. (2015) also showed that *D. longispora* is most closely related to *P. penicillatus* including its ex-type (CBS 448.69).

The genus *Diploöspora* was established by Grove (1916) with *Diploöspora rosea* as the type species. This genus was characterised by producing chains of hyaline, cylindrical to fusiform, aseptate, or 1–3-septate conidia (Tanney et al. 2015). Currently, phylogenetic analysis of the partial sequences of small subunit (SSU) ribosomal RNA gene, internal transcribed spacers (ITS), and large subunit (SSU) ribosomal RNA gene reveals that *D. rosea* is an onygenalean fungus (Tanney et al. 2015). *Diploöspora longispora* was firstly isolated from a dead leaf of *Colocasia esculenta* var. *antiquorum* in Japan (Matsushima 1976). Two varieties of *D. longispora* are available, namely *Diploöspora longispora* var. *longispora* and *Diploöspora longispora* var. *cubensis*, and the latter was originally obtained from the fallen leaves of *Leguminosae* in Cuba (Castaneda 1987). Tanney et al. (2015) presented that *D. longispora* and its varieties were most closely related to *P. penicillatus* belonging to the order *Hypocreales* and reached affinity with *Hypocreaceae*. However, apart from conidial chains, there is little morphological similarity between *P. penicillatus* and *D. longispora*, namely the penicillate conidiophores of *P. penicillatus* with their basipetal conidiogenesis versus the branched conidiophores and acropetal conidiogenesis of *D. longispora* (Tanney et al. 2015).

The genus *Paecilomyces* was introduced by Bainier (1907) with *Paecilomyces variotii* as the type species (Samson 1974). This genus was featured by verticillate conidiophores with divergent whorls of phialides, having a cylindrical or inflated base tapering to a long and distinct neck and producing typically hyaline, one-celled conidia. Phylogenetic analysis based on 18S rDNA demonstrates that *Paecilomyces* is polyphyletic across two classes (Luangsa-Ard et al. 2004). The type species, *P. variotii*, and its thermophilic relatives were placed in *Eurotiales* (*Eurotiomycetes*), while mesophilic species are in *Hypocreales* (*Sordariomycetes*) (Luangsa-Ard et al. 2004, 2005). *Paecilomyces penicillatus* was introduced by Samson in 1974, which was first isolated from rotten mushrooms. Based on the morphological characteristics, it was placed in *Eurotiales* (Samson 1974). Based on the molecular phylogenetic analysis of 18S rRNA sequences and β-tubulin, *P. penicillatus* was transferred to the order *Hypocreales* (*Sordariomycetes*) and revealed an uncertain affinity with *Hypocreaceae* (Luangsa-ard et al. 2004, 2005); however, *P. penicillatus* is still placed in *Paecilomyces* sensu stricto.

According to Hyde et al. (2020), this order comprises 14 families, including *Bionectriaceae, Calcarisporiaceae, Clavicipitaceae, Cocoonihabitaceae, Cordycipitaceae, Flammocladiellaceae, Hypocreaceae, Myrotheciomycetaceae, Nectriaceae, Niessliaceae, Ophiocordycipitaceae, Sarocladiaceae, Stachybotryaceae*, and *Tilachlidiaceae*. Recently, *Polycephalomycetaceae* was introduced for the accommodation of the fungicolous species from *Ophiocordycipitaceae* based on a concatenated matrix of six genetic markers (LSU, ITS, SSU, TEF, RPB1, and RPB2, personal communication). These hypocrealean fungi are mostly found as saprobes on decaying wood and in soil, pathogens or endophytes of plants, nematodes, and insects (Zhang et al. 2018, personal communication), as well as parasites on other fungi and lichens (Zhu and Zhuang 2013; Sun et al. 2019a). Generally, hypocrealean fungicolous taxa are the more serious, common pathogens of most cultivated mushrooms (Sun et al. 2019b). *Diploöspora longispora* and *Paecilomyces penicillatus* (*Hypocreales*) are recognised as the serious pathogens of cultivated *Morchella*, and previous studies indicated that their family ranks are uncertain (Luangsa-Ard et al. 2004; Luangsa-ard et al. 2005; Tanney et al. 2015). Additionally, Tanney et al. (2015) and our analyses presented that *D. longispora* and its variants were most closely related to several *P. penicillatus* strains (including CBS 448.69, the ex-type strain) based on an ITS BLAST query. Currently, multi-locus phylogenetic analyses and divergence time estimation have been used to clarify the higher ranking of fungal taxa (Hyde et al. 2017, 2020). This study performed phylogenetic analysis and divergence time estimation using a concatenated matrix of five genetic markers (LSU, ITS, SSU, TEF, and RPB2). Based on the results, a new family, *Pseudodiploösporeaceae*, and two new genera *Pseudodiploöspora* and *Zelopaecilomyces*, are introduced to accommodate the fungal taxa misplaced under *Diploöspora* and *Paecilomyces*, respectively. Additionally, we introduce four combinations including the fungal pathogens causing pileus rot disease and white mould disease of cultivated *Morchella*.

## Materials and methods

### Specimens, isolates, and morphological observation

Fresh specimens were collected along with the fruiting bodies of cultivated *Morchella* mushrooms in Kunming Yunnan province, Ankang, Shanxi province, Baoding, Hebei, province, Ningxia Hui Autonomous Region during the 2019 and 2021 cultivation seasons. Fruiting bodies were examined from free-hand sections using a stereomicroscope. The conidia were picked and streaked on potato extract agar (PDA) and incubated at 25°C for 7 days. A Nikon Eclipse 80i light microscope, equipped with differential interference contrast (DIC) optics, was used to capture digital images. Tarosoft (R) v.0.9.7 Image Frame Work was used to measure the morphologic structures, and Adobe Photoshop CS6 Extended version 13.0.1 software (Adobe Systems, USA) to edit the photographic plates.

For observation by SEM, each patch (0.3 × 0.3 cm) of the fresh infected and un-infected *M. sextelata* was fixed in 2.5% glutaraldehyde in 0.05 M phosphate-buffered saline (BPS, pH 7.2) at 4°C. After 24 hours, the samples were washed with deionised 0.1 M PBS for 7 min three times, then dehydrated in graded ethanol (50%,70%, 80%, 95%) for 15 min, respectively. Subsequently, the samples were dehydrated in 100% ethanol for 15 min three times and dried in a fume hood using critical point dryers (Autosamdri® 931, Tousimis, MD, USA) with CO_2_. Finally, the samples were sputter-coated with gold by an ion sputter coater (ISC150, SuPro Instruments, Shenzhen, China) with a voltage of 110 V, a frequency of 50/60 Hz, and a current of 10 mA under vacuum of lower than 1–2 Pa for 60 s. The samples were loaded onto the SEM (SU8010, Hitachi, Tokyo, Japan) and observed.

### DNA extraction, PCR amplification, and sequencing

Genomic DNA of each strain was extracted from fresh mycelium grown on PDA after 7 days of growth following the rapid “thermolysis” method described by Zhang et al. (2010). For the amplification of SSU, ITS, LSU, RPB2, and TEF1-α gene fragments, the following primer pairs: NS1/NS4 primer pair for partial small subunit ribosomal RNA gene region (SSU), ITS4/ITS5 primer pair for internal transcribed spacer gene region (ITS) (White et al. 1990), LROR/LR5 for partial large subunit rRNA gene region (LSU), 983 F/2218 R for partial translation elongation factor 1-alpha gene region (TEF-1α) (Carbone and Kohn 1999); RPB2-5 F/RPB2-7 R for partial RNA polymerase II largest subunit gene region (RPB2) (Liu et al. 1999) was used. Each PCR reaction consisted of 12.5 μl 2× Taq PCR SuperMix (TianGen Biotech Co., Beijing, China), 1 μl of each forward and reverse primer (10 μM), 0.5 μl DMSO, 3 μl DNA template, and 7 μl double sterilised water. PCR reactions were performed in a fast thermal cycler (LongGene Co., Hangzhou, China), following the protocols described by Gu et al. (2020). The PCR products were sequenced by Beijing Tianyihuiyuan Bioscience and Technology after being evaluated by electrophoresis.

### Phylogenetic analysis

SeqMan Pro v. 7.1.0 (DNASTAR Lasergene) was used to trim the low-quality bases at both ends of the raw forward and reverse reads and to assemble them. The newly obtained sequences were queried against the nuclear database of NCBI. For species delimitation, the aligned ITS sequence matrix of 58 taxa including our isolates, *Diploöspora*, and available species of *Paecilomyces* and its allied fungi, as well as *Alternaria* species (outgroup taxa) were used to construct the phylogenetic tree. The SSU, ITS, LSU, RPB2, and TEF sequences of available generic type species and reprehensive of *Hypocreales* and representative species of all accepted *Hypocaceae* from recent studies (Sun et al. 2017) were employed for multi-locus phylogenetic analysis. *Gelasinospora tetrasperma, Neurospora crassa* and *Sordaria fimicola* were chosen as the outgroup taxa. The alignments were generated by using MAFFT version 7.03 with the Q-INS-I strategy (Katoh and Standley 2013). Conserved blocks were selected from the initial alignments with Gblocks 0.91 b (Castresana 2000). The best nucleotide substitution model for each gene was determined by using jModeltest2.1.1 (Darriba et al. 2012). GTR+G + I was estimated as the best-fit model for ITS; RPB2, TN93 + G was estimated as the best-fit model for SSU; and LSU, TN93 + G + I as the best-fit model for TEF-1α under the output strategy of BIC. The multi-locus phylogenetic analyses included 1403 characters for SSU, 607 characters for ITS, 893 characters for LSU, 1044 characters for RPB2, and 907 characters for TEF. All characters were weighted equally, and gaps were treated as missing characters.

Maximum likelihood (ML) analyses were performed by RAxML2.0 (Edler et al. 2021), using the GTR+GAMMA+I model. The maximum likelihood bootstrap proportions (MLBP) were determined using 1000 replicates. Bayesian inference (BI) analyses were conducted with MrBayes v3.2.7 (Ronquist et al. 2012). Metropolis-coupled Markov Chain Monte Carlo (MCMC) searches were calculated for 10,000,000 generations, sampling every 100th generation with the best-fit model for each gene. Two independent analyses with six chains each (one cold and five heated) were carried out until the average standard deviation of the split frequencies dropped below 0.01. The initial 25% of the generations of MCMC sampling were discarded as burn-in. The refinement of the phylogenetic tree was used for estimating Bayesian inference posterior probability (PP) values. The tree was viewed in FigTree v1.4 (Rambaut 2012), and values of maximum likelihood bootstrap proportion (MLBP) greater than 50% and Bayesian inference posterior probabilities (BIPP), greater than 95% at the nodes, are shown along branches.

### Relative divergence time estimation

Molecular dating analysis was performed using BEAST v1.10.4 (Suchard et al. 2018). The aligned data were partitioned for each SSU, ITS, LSU, RPB2, and TEF1 dataset, and these were loaded to BEAUti v1.10.4. to prepare the XML file. The data partitions were set with unlinked substitution and clock models to independently estimate each gene partition. Taxa sets were developed for each calibration of the common ancestor nodes, associated with the most recent common ancestor (TMRCA). The *Hypocreales* crown with a normal distribution (mean = 216, SD = 27.5, with 97.5% of CI = 269 MYA). Calibration of the core *Clavicipitaceae*, using a normal distribution (mean = 133.7, SD = 20.8, with 97.5% of CI = 174.5 MYA). Calibration of the Ophiocordyceps crown, using an exponential distribution (offset = 100, mean = 27.5, with 97.5% CI of 200 MYA) (Samarakoon et al. 2016; Hyde et al. 2017). The Yule process tree prior was used to model the speciation of nodes in the topology with a randomly generated starting tree. The analyses were performed for 100 million generations, with sampling parameters every 1000 generations. The effective sample sizes were checked in Tracer v.1.7.2 and the acceptable values are higher than 200. The first 10,000 trees (10%) representing the burn-in phase were discarded based on Tracer v.1.7.2, and 90,000 trees were combined in LogCombiner v1.10.4. The maximum clade credibility (MCC) tree was given by summarised data and estimated in TreeAnnotator v1.10.4. The molecular dating tree was viewed in FigTree v1.4 (Rambaut 2012). In the MCC tree, node bars indicate 90% confidence intervals for the divergence time estimates.

## Results

### Phylogenetic analyses

**Figure 1.**
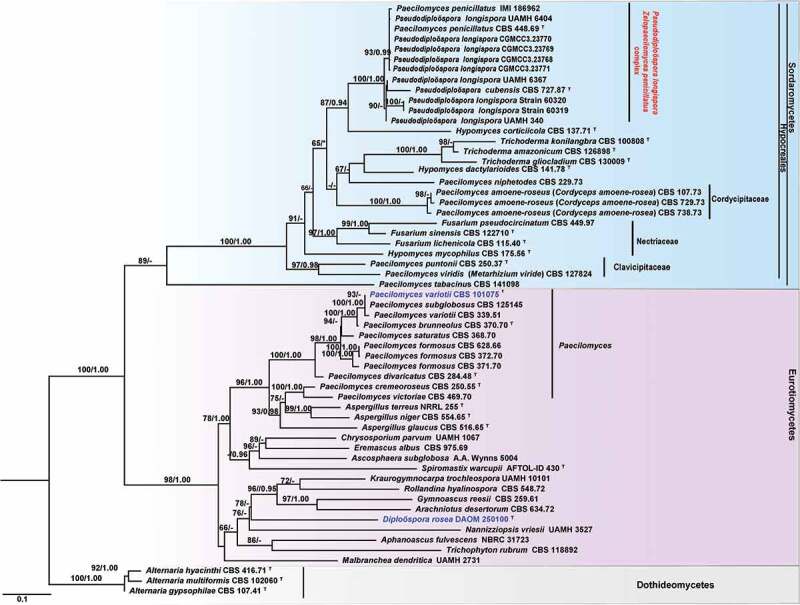
Phylogenetic analysis of *Diploöspora longispora* and *Paecilomyces penicillatus* based on ITS data set. The tree is rooted with three *Alternaria* species (*Dothideomycetes*). Bootstrap values higher than 50% from RAxML (BSML) (left) are given above the nodes. Bayesian posterior probabilities greater than 0.95 are indicated (BYPP) (right). Hyphens indicate bootstrap values less than 50% or Bayesian posterior probability values lower than 0.90. ^T^ indicates the type. The type species of *Diploöspora* and *Paecilomyces* are in blue.

The phylogenetic trees showed that generic type species of *Diploöspora rosea* (DAOM 250100) and *Paecilomyces variotii* (CBS 101075) were positioned in the class *Eurotiomycetes*, while isolates of *Diploöspora longispora* and *Paecilomyces penicillatus* were placed in the class *Sordariomycetes* (Figure 1). Within *Sordariomycetes, D. longispora* (UAMH 340, UAMH 6404, UAMH 6367, strain 60319, and strain 60320), *D. longispora* var. *cubensis* (CBS 727.87), and *P. penicillatus* (CBS 448.69, IMI 186962) clustered together with maximum support (MLBP/BIBP = 100%/1.00, Figure 1). In the phylogenetic tree, those fungi also showed affinities with hypocrealean fungi, especially close to *Hypomyces corticiicola* (MLBP/BIBP = 100%/1.00, Figure 1).

**Figure 2.**
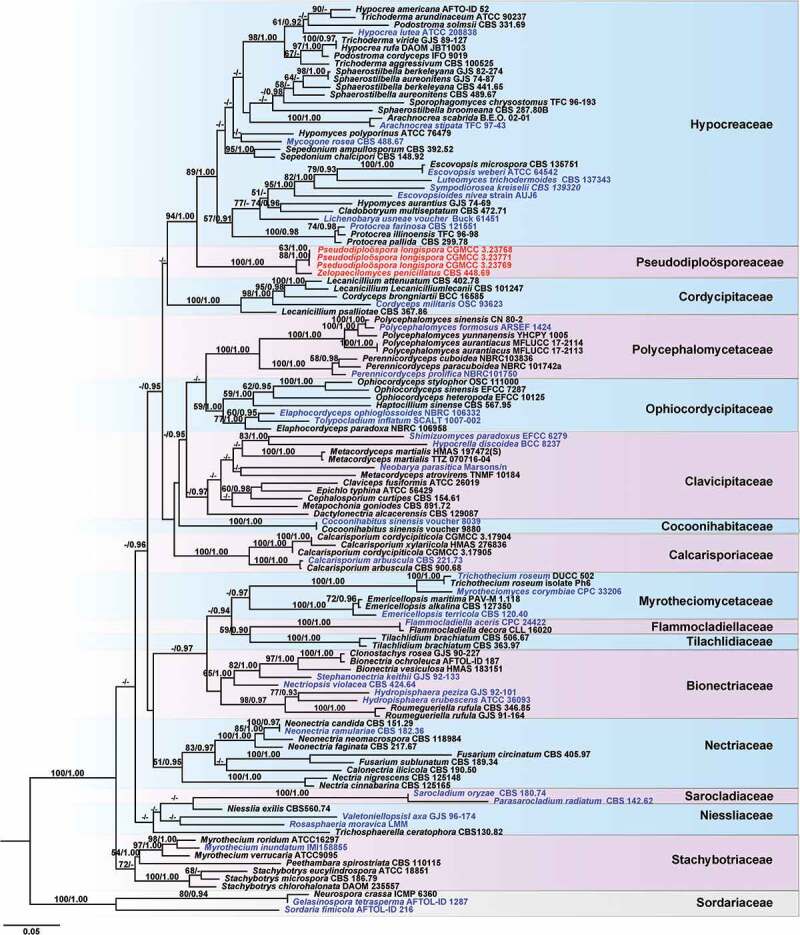
Multi-locus phylogenetic analysis of *Hypocreales* based on a combined SSU, ITS, LSU, TEF, and RPB2 data set. The tree is rooted with *Gelasinospora tetrasperma, Neurospora crassa,* and *Sordaria fimicola*. Bootstrap values higher than 50% from RAxML (BSML) (left) are given above the nodes. Bayesian posterior probabilities greater than 0.90 are indicated (BYPP) (right). Hyphens indicate bootstrap values less than 50% or Bayesian posterior probability values lower than 0.90. The generic type species are in blue.

To determine the family placement of *Diploöspora longispora* and *Paecilomyces variotii*, a phylogenetic tree was constructed with a sequence matrix of five alignment files including SSU (1043 bp), ITS (607), LSU (884 bp), EF1-α (907 bp), and RPB2 (1044 bp) sequence data (a total of 4853 characters) from 111 taxa of *Hypocreales* and three outgroup taxa (*Gelasinospora tetrasperma, Neurospora crassa*, and *Sordaria fimicola*; Figure 2). The phylogenetic tree well explained the phylogenetic relationship within *Hypocreales*. There were 15 clades formed in *Hypocreales* corresponding to the families *Bionectriaceae, Calcarisporiaceae, Clavicipitaceae, Cordycipitaceae, Flammocladiellaceae, Cocoonihabitaceae, Hypocreaceae, Nectriaceae, Niessliaceae, Ophiocordycipitaceae, Polycephalomycetaceae, Sarocladiaceae, Stachybotryaceae*, and the clade comprising *Diploöspora longispora* and *Paecilomyces penicillatus. Diploöspora longispora* and *P. penicillatus* are phylogenetically distinct from the type species of their respective genera and are better accommodated in as-yet undescribed genera. They are hereafter referred to as *Pseudodiploöspora longispora* and *Zelopaecilomyces penicillatus*, respectively, and formally described below. *Pseudodiploöspora longispora* and *Z. penicillatus* form a strongly supported (MLBP/BIBP = 100%/1.00; Figure 2) distinct clade sister to *Hypocreaceae* with robust support (MLBP/BIBP = 94%/1.00; Figure 2). Based on its phylogenetic distinction from *Hypocreaceae*, this clade is described below as *Pseudodiploösporeaceae* fam. nov.

### Relative divergence time estimation

**Figure 3.**
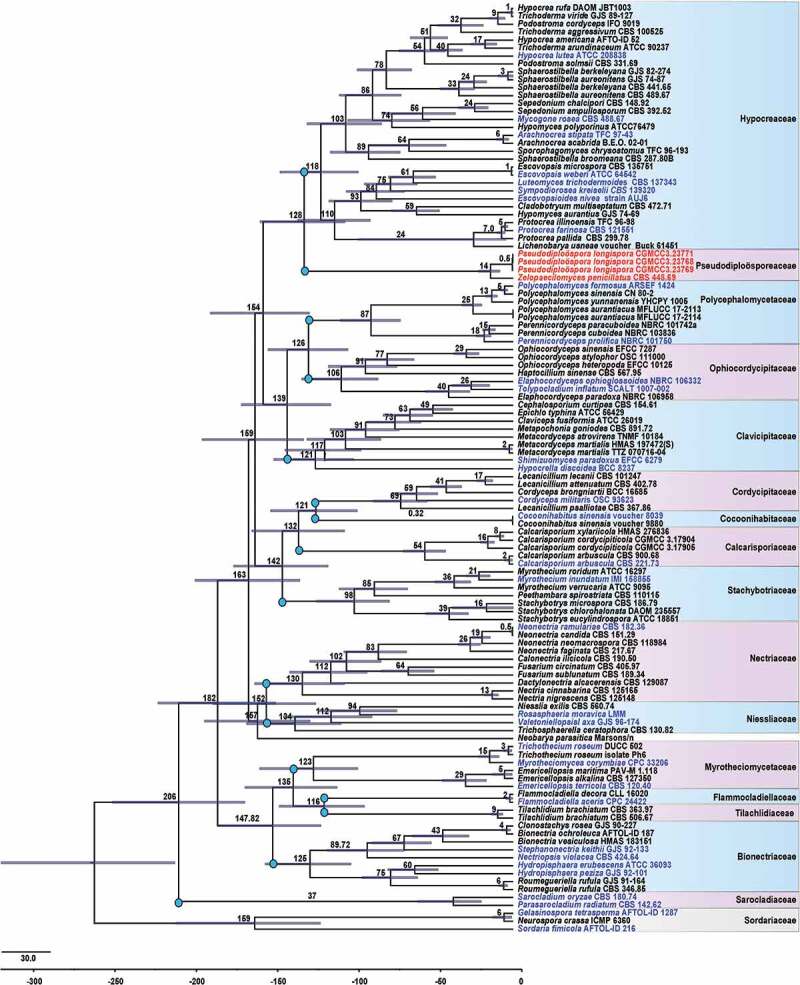
The MCC tree of *Hypocreales*, including some representative strains of *Sordariales*, was obtained from a Bayesian approach (BEAST). Bars correspond to the 95% highest posterior density (HPD) intervals. The fossil minimum age constraints and second calibrations used in this study are marked with green dots. The divergence time of orders is marked in purple dots and families with blue dots. The generic type species are in blue.

According to the divergence time estimates, the crown age of *Hypocreales* is around 206 (165–246) MYA (Figure 3). Based on our analysis, *Sarocladiaceae* was the earlier diverged family in *Hypocreales*, which diverged from other hypocrealean fungi at approximately 159 MYA. *Flammocladiellaceae* and *Tilachlidiaceae* were the youngest families within *Hypocreales*, which diverged from each other about 116 MYA. In general, the divergence time for the currently accepted 15 families is within the range of 116–159 MYA, suggesting that a family can at best be as young as 116 MYA. In the MCC tree, our newly generated *Pseudodiploösporeaceae* diverged from *Hypocreaceae* at about 129 MYA, falling within the temporal band of families.

## Taxonomy

*Pseudodiploösporeaceae* Jing Z. Sun, X.Z. Liu & H.W. Liu, fam. nov.

Fungal name: FN 571280

*Etymology*: *Pseudodiploöspor-*, from the genus name *Pseudodiploöspora*, and -aceae is the family suffix

*Type genus: **Pseudodiploöspora*** Jing Z. Sun, X.Z. Liu & H.W. Liu, gen. nov.

*Description*: Saprobic or fungicolous; **Sexual morph**: Undetermined. **Asexual morph**: *Colonies* on natural substrate effuse, whitish. *Mycelia*, superficial or immersed; *Hyphae* branched, septate, hyaline. *Conidiophores* micronematous to macronematous, mononematous, penicillate. *Conidiogenous cells* sympodial, acropetal, basipetal, hyaline. *Conidia* cylindrical, ellipsoidal, limoniform, solitary, or catenate in simple or branched chains. *Ramoconidia* cylindrical or fusiform, aseptate or septate, truncate at the base, with terminal scars.

*Note*: Both phylogenetic analysis and molecular clock evidence based on SSU, ITS, LSU, TEF, and RPB2 sequence data support *Pseudodiploösporeaceae* as a sister group of *Hypocreaceae*. The MCC tree estimates that *Pseudodiploösporeaceae* split from *Hypocreaceae* around 129 MYA, falling within the temporal band of families (50–150 MYA) (Hyde et al. 2017). Therefore, *Pseudodiploösporeaceae* is introduced as a new family within *Hypocreales*, to accommodate *Pseudodiploöspora* and *Zelopaecilomyces.*

*Pseudodiploöspora* Jing Z. Sun, X.Z. Liu & H.W. Liu, gen. nov.

Fungal name: FN 571281

*Etymology*: *pseudo*, in Latin, meaning “false or spurious thing”, referring to members of this genus being morphologically similar to *Diploöspora* but phylogenetically distinct to the *Diploöspora* species

*Type species*: *Pseudodiploöspora longispora* (Matsush.) Jing Z. Sun, X.Z. Liu & H.W. Liu, comb. nov.

*Description*: Saprobic or fungicolous; **Sexual morph**: Undetermined. **Asexual morph**: *Colonies* on natural substrate effuse, whitish. *Mycelia*, superficial or immersed; *Hyphae* branched, septate, hyaline. *Conidiophores* micronematous to macronematous, aseptate or septate. *Conidiogenous cells* sympodial, acropetal, hyaline. *Conidia* cylindrical, ellipsoidal, fusiform, catenate in simple or branched chains, hyaline. *Ramoconidia* cylindrical or fusiform, truncate at the base, with terminal scars, hyaline.

*Note*: The genus *Diploöspora* was established by Grove (1916) with *Diploöspora rosea* as the type species. Phylogenetic evidence supported that *D. rosea* is an onygenalean fungus within *Eurotiomycetes* (Tanney et al. 2015). *Diploöspora longispora* and its two variants, *D. longispora* var. *longispora* and *D. longispora* var. *cubensis*, were isolated originally from the fallen leaves (Matsushima 1976; Castañeda 1987). Based on an ITS BLAST query, Tanney et al. (2015) proposed that *D. longispora* and its varieties belong to the order *Hypocreales*, and reached affinity with *Hypocreaceae*. While, our phylogenetic analysis based on the ITS sequence data also showed strains of *D. longispora* (UAMH 340, UAMH 6404, UAMH 6367, strain 60,319, and strain 60,320), and *D. longispora* var. *cubensis* (CBS 727.87, IMI 186962) grouped with strong support (MLBP/BIBP = 100%/1.00, Figure 1) in *Sordariomyetes* rather than in *Eurotiomyetes*. In our multi-locus phylogenetic tree, those taxa clustered in a distinct clade within *Hypocreales* but do not belong to *Hypocreaceae* (Figure 2), representing a new genus rank. Morphologically, despite those taxa and *Diploöspora* producing conidial chains, they are distinct from *Diploöspora* in acropetal conidiogenesis and the shape and size of conidia. *Pseudodiploöspora* is therefore introduced herein to accommodate those species misplaced in *Diploöspora*.

*Pseudodiploöspora fungicola* (R.F. Castañeda) Jing Z. Sun, X.Z. Liu & H.W. Liu, comb. nov.

Fungal name: FN 571282

*Basionym*: *Diploöspora fungicola* R.F. Castañeda, Fungi Cubenses II: 4 (1987)

*Type*: INIFAT C86/132 (Holotype)

*Description*: See the original description in Castañeda Ruiz, R.F. (1987), Fungi Cubenses II, p. 22

*Substrate*/*Host*: On dead basidioma of *Auricularia*

*Distribution*: Cuba

*Note*: There is no available sequence of *Diploöspora fungicola*, and its morphological characters are highly similar to *Pseudodiploöspora longispora*. Additionally, this species colonised the basidioma of *Auricularia*, which suggests a similar fungicolous ecology relating it to *Pseudodiploöspora longispora* (Castañeda Ruiz, R.F. 1987).

**Figure 4.**
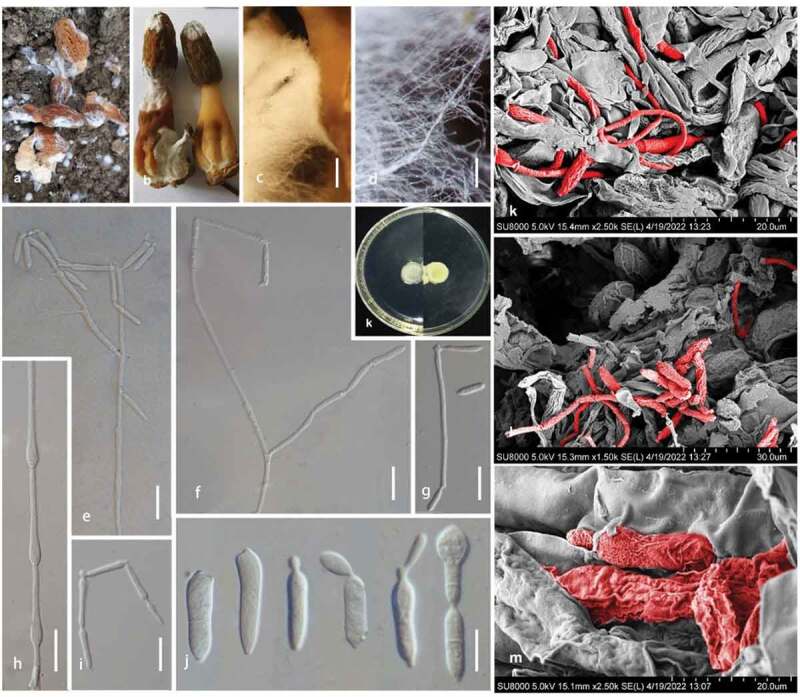
***Pseudodiploöspora longispora*** (CGMCC 3.23768). (a, b) *Pseudodiploöspora longispora* and its host fungus (*Morchella sextelata*). (c, d) Mycelia on a fruiting body of *M. sextelata*. (e–h) Conidiophores with conidia. (i) Conidiogenous cell. (j) Conidiogenous scars and conidia. (k–m) Hypha and conidia of *P. longispora* (in red) across the fruiting body of *M. sextelata*. Scale bars: c = 200 μm; d = 100 μm; e-g, i = 25 μm; h, j = 10 μm.

**Figure 5.**
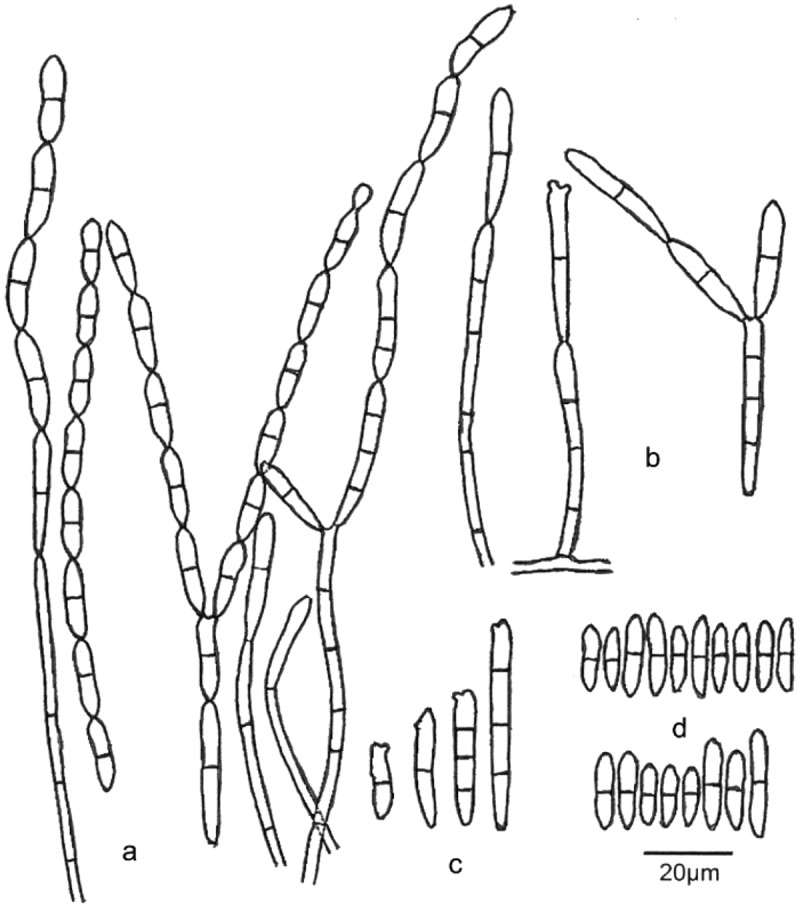
***Pseudodiploöspora longispora*** (*Diploöspora longispora*, INIFAT C87/58, Holotype!). (a, b) Conidiophores with conidia. (). Conidiogenous cell. (d) Conidiogenous scars and conidia. Scale bars = 20 μm. Redrawn from Matsushima (1975).

*Pseudodiploöspora longispora* (Matsush.) Jing Z. Sun, X.Z. Liu & H.W. Liu, comb. nov. Figures 4 and 5

Fungal name: FN 571283

*Basionym*: *Diploöspora longispora* Matsush., Icones Microfungorum a Matsushima lectorum: 61 (1975) Figure 5

*Synonym*: *Diploöspora longispora* var. *longispora* Matsush., Icones Microfungorum a Matsushima lectorum: 61 (1975)

*Type*: INIFAT C87/58 (Holotype)

*Description*: See the original description in Matsushima, T. (Matsushima and Matsushima 1976), Icones Microfungorum a Matsushima Lectorum, p. 61.

*Substrate*/*Host*: On dead leaf of *Colocasia esculenta* var. *antiquorum*, Japan (Matsushima and Matsushima 1976). On the fruiting body of cultivated *Morchella* spp., China (CGMCC 3.23768, CGMCC 3.23769, CGMCC 3.23770, CGMCC 3.23771). Skin and foot, Canada (UAMH 340) Canada (https://www.uamh.ca/index.html)

*Distribution*: Japan, China

**Figure 6.**
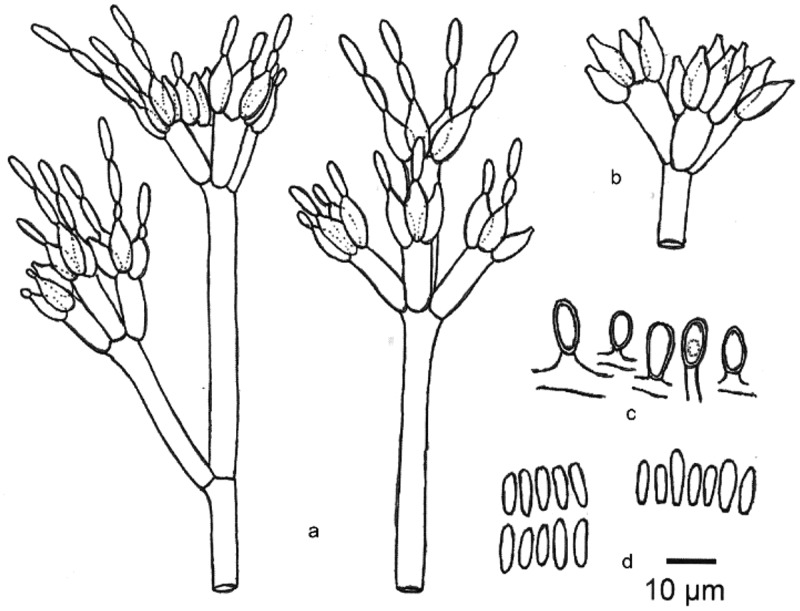
***Zelopaecilomyces penicillatus*** (CBS 448.69, ex-type strain). (a) Conidiophores with conidia (CBS 448.69). (b) Phialides (type *Spicaria penicillate*). (c) Chlamydospores. (d) Conidia. Scale bars = 10 μm. Redrawn from Samson (1974).

*Note*: *Diploöspora longispora* was first isolated from a dead leaf of *Colocasia esculenta* var. *antiquorum* in Japan (Matsushima and Matsushima 1976). We introduce a new combination of *Pseudodiploöspora longispora* to accommodate *D. longispora*. Both analyses in Tanney et al. (2015) and this study suggested that *Pseudodiploöspora longispora* is most closely related to *Paecilomyces penicillatus*. However, the latter differs from *Pseudodiploöspora longispora* in the penicillate conidiophore, and basipetal conidiogenesis, as well as the shape and size of conidia (Figures 5 and 6). We did not treat *Paecilomyces penicillatus* as a synonym of *Pseudodiploöspora longispora* herein because of the great morphological differences.

*Pseudodiploöspora cubensis* (R.F. Castañeda) Jing Z. Sun, X.Z. Liu & H.W. Liu, comb. nov. Figures 4 and 5

Fungal name: FN 5712997

*Synonym*: *Diploöspora longispora var. cubensis* R.F. Castañeda, Fungi Cubenses II: 5 (1987)

*Type*: CBS 727.87 (ex-type strain)

*Description*: See the original description in Castañeda (1987), Fungi Cubenses II.:1-22

*Substrate*/*Host*: On fallen leaves of Leguminosae: Cuba (Castaneda 1987). On porcupine dung in a cave (Including UAMH 6367, UAMH 6404) (https://www.uamh.ca/index.html)

*Distribution*: Cuba

*Note*: *Pseudodiploöspora cubensis* was originally obtained from the fallen leaves of Leguminosae in Cuba (Castaneda 1987). The ITS sequence of CBS 727.87 is 96% similar to *Pseudodiploöspora longispora* (identities, 514/537, gaps, 5/537). Additionally, regarding the ellipsoidal conidia of *Pseudodiploöspora cubensis* against cylindrical or ramoconidia of *P. longispora*, as well as the original isolation resource and location, we introduce a new combination of *Pseudodiploöspora cubensis* to accommodate *D. longispora* var. *cubensis*.

*Pseudodiploöspora zinniae* (Matsush.) Jing Z. Sun, X.Z. Liu & H.W. Liu, comb. nov.

Fungal name: FN 571284

*Basionym*: *Diploöspora zinniae* Matsush., Matsushima Mycological Memoirs 2: 8 (Matsushima 1981)

*Type*: MBT 70959

*Description*: See the original description in Matsush. (1981), Matsushima Mycological Memoirs 2, p. 8

*Substrate/Host*: Seed of *Zinnia elegans*

*Distribution*: Japan

*Note*: There is no available sequence of *Diploöspora zinnia*, and we transfer this fungus to *Pseudodiploöspora* based on its sympodial, acropetal conidiogenesis, and the cylindrical-fusiform conidia (Matsushima 1981).

*Zelopaecilomyces* Jing Z. Sun, X.Z. Liu & H.W. Liu, gen. nov.

Fungal name: FN 571285

*Etymology*: *Zelo*, meaning “emulation”, refers to members of this genus being morphologically similar to *Paecilomyces* but phylogenetically distinct from the true *Paecilomyces* species

*Type species*: *Zelopaecilomyces penicillatus* (Samson) Jing Z. Sun, X.Z. Liu & H.W. Liu, comb. nov.

*Description*: Saprobic or fungicolous; Sexual morph: **Undetermined**. Asexual morph: Colonies on natural substrate effuse, whitish. Mycelia, superficial or immersed; Hyphae branched, septate, hyaline. Conidiophores mononematous, penicillate, with whorls of phialides. Phialides cylindrical, basal portion with distinct neck. Conidiogenous cells basipetal, hyaline. Conidia cylindrical, ellipsoidal, solitary, or catenate in simple or in chains, aseptate, truncate at the base, with terminal scars. Chlamydospores produced submerged in the agar, single, ellipsoidal to pyriform, aseptate.

*Note*: The genus *Paecilomyces* was introduced by Bainier (1907) with *Paecilomyces variotii* as the type species. The type species, *P. variotii*, and its thermophilic relatives were placed in *Eurotiales* (*Eurotiomycetes*), while entomopathogenic mesophilic species were placed in *Hypocreales* (*Sordariomycetes*) under the genus *Isaria* but did not include *Paecilomyces penicillatus* (Luangsa et al. 2004; Luangsa-Ard et al. 2005). Those taxa placed in *Isaria* were accepted in *Samsoniella* (*Hypocreales, Cordycipitaceae*) (Mongkolsamrit et al. 2018). Our phylogenetic analyses showed that *Z. penicillatus* (CBS 448.69, ex-type strain) was positioned in *Pseudodiploösporeaceae* (Figure 2). Despite a more than 99% similarity of the SSU (identities, 1589/1590, gap, 1/1590) and ITS sequence (identities, 502/505; gaps, 3/505 gaps) between *Z. penicillatus* (CBS 448.69) and *Pseudodiploöspora loogispora*, respectively. The ITS of *Z. penicillatus* is 4% different from *Pseudodiploöspora cubensis* (identities, 471/477; gaps, 6/477). Additionally, *Z. penicillatus* differs from both *P. cubensis* and *P. loogispora* in having penicillate conidiophores and basipetal conidiogenesis. Herein, we introduce *Zelopaecilomyces* for the accommodation of *P. penicillatus* based on its morphological distinctions.

*Zelopaecilomyces penicillatus* (Höhn.) Jing Z. Sun, X.Z. Liu & H.W. Liu, comb. nov. Figure 6

Fungal name: FN 571286

*Basionym*: *Paecilomyces penicillatus* (Höhn.) Samson, Studies in Mycology 6: 72 (1974)

*Spicaria penicillata* Höhn., Annales Mycologici 2 (1): 56 (1904)

*Type*: ex-type strain CBS 448.69

*Description*: See the original description in Samson (1974), *Paecilomyces* and some allied *Hyphomycetes*, Studies in Mycology, 6, p.72

*Substrate*/*Host*: Peridia of *Arcyria cinerea*, rotting *Agaricus bisporus* mushroom

*Distribution*: Austria, Belgium

*Note*: *Zelopaecilomyces penicillatus* (*Spicaria penicillata*) was introduced by Höhnel (1904) based on its morphological characteristics. It was first isolated from the peridia of the myxomycete *Arcyria cinerea* and later isolated from a rotten *Agaricus bisporus* mushroom, with the resulting strain (CBS 448.69) treated as the ex-type strain (Samson 1974). Herein, we introduce a new combination, *Zelopaecilomyces penicillatus,* in consideration of the distinct phylogenetic position and morphological features of *P. penicillatus*.

## Discussion

A combination of phylogenetic analyses and divergence time estimation has been widely used in solving the classification schemes and higher ranking of taxa (Hyde et al. 2017). According to this polyphasic approach, a large number of taxonomic positions of fungi have been refined (Hyde et al. 2020; He et al. 2022). Hyde et al. (2020) gave an update of *Sordariomycetes* based on phylogenetic analyses and divergence time estimation. According to their results, *Hypocreales* contained 14 families: *Bionectriaceae, Calcarisporiaceae, Clavicipitaceae, Cocoonihabitaceae, Cordycipitaceae, Flammocladiellaceae, Hypocreaceae, Myrotheciomycetaceae, Nectriaceae, Niessliaceae, Ophiocordycipitaceae, Sarocladiaceae, Stachybotryaceae*, and *Tilachlidiaceae*. Both our multi-locus phylogeny and divergence time evidence reveal the proposed natural classification of *Hypocreales*. Multi-locus phylogeny reals a family rank for *Pseudodiploösporeaceae* because its taxa formed a strongly supported and distinct clade sister to *Hypocreaceae*. Hyde et al. (2017) introduced a temporal banding for *Ascomycota*, and time ranges of 150–250 MYA and 50–150 MYA were recommended as the boundary for orders and families, respectively. Our MCC results presented that the crown age of *Hypocreales* is around 206 (165–246) MYA (Figure 3), which concurs with the previous results (Hyde et al. 2017, 2020). Within *Hypocreales*, the divergence time for currently accepted families is within the range of 116–159 MYA suggesting that a family can at best be as young as 116 MYA in *Hypocreales*. Divergence time showed that the family *Pseudodiploösporeaceae* divorced from *Hypocreaceae* about 129 MYA, falling within the temporal band of families. Additionally, *Polycephalomycetaceae* was recently introduced as a new family based on a concatenated matrix of six genetic markers (SSU, ITS, LSU, RPB1, RPB2, and TEF) (personal communication), both our phylogenetic tree and MCC tree also support its family rank in *Hypocreales* herein. Vu et al. (Vu et al. 2019) proposed a taxonomic threshold predicted for filamentous fungal identification, and 88.5% similarity of ITS barcodes was suggested for family rank. A BLAST querying the ITS sequence of species from *Pseudodiploösporeaceae* presented less than 89% similarity against that species from *Hypocreales*, which also supported distinct family rank for *Pseudodiploösporeaceae*.

The taxonomic position of *Diploöspora* Grove was confirmed as a member of *Eurotiomycetes* by re-examination of its generic type species *D. rosea* (Tanney et al. 2015). Several species including *Pseudodiploöspora longispora* (previously known as *Diploöspora longispora*) and *Pseudodiploöspora cubensis* (previously known as *Diploöspora longispora* var. *cubensis*) placed previously in *Diploöspora* were shown an affinity for Hypocrealean fungi (*Sordariomycetes*) based on the phylogenetic analysis (Luangsa-Ard et al. 2004, 2005; Tanney et al. 2015). Our phylogenetic analysis also supported that *P. longispora* and *P. cubensis* were more closely related to *Hypocreaceae* (Figure 1–2). In our multi-locus phylogenetic tree, those taxa clustered in a distinct clade within *Hypocreales* but were outside of the core *Hypocreaceae* (Figure 2), representing a new family and subsequent genera. *Pseudodiploöspora* is therefore introduced herein to accommodate those species misplaced in *Diploöspora* concerning the original nomenclature. *Pseudodiploöspora* is distinct from *Diploöspora* in having head-to-tail (acropetal) arrays of conidiogenesis against the latter of tail-to-head (basipetal) arrays of conidiogenesis. Additionally, the conidia of *Pseudodiploöspora* are longer but more slender than that of *Diploöspora* (Tanney et al. 2015). We introduce *P. longispora* and *P. cubensis* for accepting *D. longispora* and *D. longispora* var. *cubensis* regarding the 95.66% similarity of ITS sequence between *P. cubensis* (CSB 727.877) and other *P. longispora* isolates. Despite lacking molecular data on *Diploöspora fungicola* and *Diploöspora zinnia*, we enrolled them in *Pseudodiploöspora* according to the morphological features in the original description. *Diploöspora coprophilia* with phialides and producing subglobose conidia is unlikely to be related to *Diploöspora rosea* (Tanney et al. 2015) and *Pseudodiploöspora longispora*. Its taxonomic position needs to be further demonstrated. It was suggested that *Diploöspora indica* producing brown conidiophores may be better placed in *Parapleurotheciopsis* but not in *Diploöspora* (Tanney et al. 2015). We also excluded *D. indica* from *Pseudodiploöspora* in consideration of the brown conidiophore of the fungus.

Both analyses by Tanney et al. (2015) and this study presented that *P. longispora* is most closely related to *Z. penicillatus* (CBS 448.86) (Figure1–2). Vu et al. (2019) proposed a 99.6% similarity of ITS barcode for a species taxonomic threshold. When comparing the similarity of the ITS sequence, *Z. penicillatus* presented less than 98.63% similarity to that of *P. longispora*, and showed less than 94.3% similarity to that of *P. cubensis* (KT279809, CBS 727.87, ex-living type, previously known as *Diploöspora longispora* var. *cubensis*), respectively. There were no available EF1-α and RPB2 sequences in GenBank. We did not compare the similarity of EF1-α and RPB2 sequences. However, *P. penicillatus* differs from the latter in having penicillate conidiophores and basipetal conidiogenesis. Herein, we introduce a new genus, *Zelopaecilomyces,* for the accommodation of *P. penicillatus*.

The taxonomic position of *Paecilomyces* was revised and refined by phylogenetic analyses, habitats, host range, etc. (Luangsa-Ard et al. 2004, 2005). The entomopathogenic mesophilic species were placed in the class *Sordariomycetes* belonging to *Hypocreales* (Luangsa et al. 2004, 2005). Generally, those taxa were placed in *Isaria*, which were accepted by a new genus *Samsoniella* (*Hypocreales, Cordycipitaceae*) currently (Mongkolsamrit et al. 2018). Our phylogenetic analyses presented that *Z. penicillatus* (CBS 448.69, ex-type strain) was positioned in *Pseudodiploösporeaceae* (Figure 2), which was owing to a higher similarity of the SSU and ITS sequence between *P. penicillatus* and *D. longispora*. However, previous phylogenetic studies have evidenced that the SSU and ITS sequence data alone are insufficient to provide good resolution in most of the groups in *Sordariomycetes*(Hyde et al. 2020). Morphologically, *Z. penicillatus* differs from *P. longispora* by penicillate conidiophore, basipetal conidiogenesis, and the shape and size of conidia (Figure 5 and 6). Additionally, the divergence time revealed that *Z. penicillatus* diverged from *P. longispora* about 14 MYA (Figure 3). Tanney et al. (2015) thought that *P. longispora* and *Z. penicillatus* may be two extremes of a continuum, However, we treat *P. penicillatus* as a distinct species other than a synonym of *D. longispora* not only relying on morphological differences but also following the divergence time.

Both *P. longispora* and *Z. penicillatus* were originally isolated from decaying leaves and found on the rotten mushroom successively (Samson 1974; Matsushima 1976; Castaneda 1987). He et al. (2017) identified *Z. penicillatus* (as *Paecilomyces penicillatus*) as the causing agent of the white mould disease of cultivated *Morchella* only relying on ITS phylogenetic analysis but lacking morphological evidence. Liu et al. (2019) reported *P. longispora* (as *D. longispora*) infecting cultivated *Morchella*, resulting in pileus rot but not offered the typical morphological feature of *P. longispora*. The phylogenetic analyses in both Tanney et al. (2015) and this study revealed that ITS and SSU are unable to adequately distinguish *D. longispora* and *Z. penicillatus*, but our study offered robust morphological evidence on the taxonomy of *P. longispora* and *Z. penicillatus*. Since *P. longispora* has been reported as a serious fungal pathogen (Hyde et al. 2017; Liu et al. 2018), reliably taxonomic information will facilitate tracing the origin and understanding of pathogenesis.
